# Effect of subchorionic hematoma on first-trimester maternal serum free β-hCG and PAPP-A levels

**DOI:** 10.61622/rbgo/2024rbgo66

**Published:** 2024-07-26

**Authors:** Arife Akay, Yıldız Akdaş Reis, Büşra Şahin, Asya Kalaycı Öncü, Mehmet Obut, Cantekin İskender, Şevki Çelen

**Affiliations:** 1 Etlik Zübeyde Hanım Maternity and Women’s Health Teaching and Research Hospital Department of Obstetrics and Gynecology Ankara Turkey Etlik Zübeyde Hanım Maternity and Women’s Health Teaching and Research Hospital, Department of Obstetrics and Gynecology, Ankara, Turkey.; 2 Bingöl State Hospital Department of Obstetrics and Gynecology Bingöl Turkey Bingöl State Hospital, Department of Obstetrics and Gynecology, Bingöl, Turkey.; 3 Akçakoca Public Hospital Department of Obstetrics and Gynecology Düzce Turkey Akçakoca Public Hospital, Department of Obstetrics and Gynecology, Düzce, Turkey.; 4 Erciş Şehit Rıdvan Çevik Public Hospital Van Turkey Erciş Şehit Rıdvan Çevik Public Hospital, Van, Turkey.; 5 Diyarbakir Gazi Yasargil Training and Research Hospital Department of Perinatology Diyarbakır Turkey Diyarbakir Gazi Yasargil Training and Research Hospital, Department of Perinatology, Diyarbakır, Turkey.; 6 Etlik City Hospital Department of Perinatology Ankara Turkey Etlik City Hospital, Department of Perinatology, Ankara, Turkey.

**Keywords:** Abortion, threatened, Chorionic gonadotropin, Subchorionic hematoma, Pregnancy-associated plasma protein-A, Pregnancy trimester first, Aneuploidy, Biomarkers

## Abstract

**Objective:**

This study aimed to investigate the effects of the presence of subchorionic hematoma (SH) in early pregnancies with threatened miscarriage (TM) on levels of first-trimester maternal serum markers, pregnancy-associated plasma protein-A (PAPP-A), and free β-human chorionic gonadotropin (β-hCG) levels.

**Methods:**

The data of TM cases with SH in the first trimester between 2015 and 2021 were evaluated retrospectively. The data of age and gestational age-matched TM cases without SH were also assessed to constitute a control group. Demographic characteristics, obstetric histories, ultrasonographic findings, and free β-hCG and PAPP-A levels of the groups were compared.

**Results:**

There were 119 cases in the study group and 153 cases in the control group. The median vertical and longitudinal lengths of the SH were 31 mm and 16 mm. The median age of both groups was similar (p=0.422). The MoM value of PAPP-A was 0.088 (.93) in the study group and 0.9 (0.63) in the control group (p=0.519). Similarly, the MoM value of free β-hCG was 1.04 (0.78) in the study group and 0.99 (0.86) in the control group (p=0.66). No significant relationship was found in the multivariate analysis between free β-hCG MoM, PAPP-A MoM, age, gravida, and vertical and longitudinal lengths of the hematoma (p>0.05).

**Conclusion:**

The level of PAPP-A and free β-hCG were not affected by the SH. Therefore, these markers can be used reliably in TM cases with SH for the first-trimester fetal aneuploidy screening test.

## Introduction

Subchorionic hematoma (SH) is one of the ultrasonographic findings in cases with threatened miscarriage (TM) furthermore its etiology is still unknown.^(1)^Many studies have shown that SH is associated with an increased risk of poor obstetric outcomes such as pregnancy loss, preterm birth (PB), fetal growth restriction (FGR), pregnancy-induced hypertensive diseases (PIHD), and placental abrasion (PA).^(2-5)^SH is generally a half-moon-shaped, anechoic, or hypoechoic area accompanied by a normal gestational sac, and is thought to cause placental dysfunction due to the separation of the chorionic membranes from the uterine wall.^(6)^It is also thought to be the result of subchorionic hemorrhage that occurs before the development of placental adaptations to cope with oxidative stress.^(7)^Studies on the etiology of poor obstetric outcomes draw attention to the importance of normal placental development.^(8)^However, SH has been associated with increased pregnancy loss^(9)^ and poor obstetric outcomes.^(10)^

In the first trimester of pregnancy, pregnancy-associated plasma protein-A (PAPP-A) and free β-human chorionic gonadotropin (β-hCG) are placentally produced biochemical markers.^(11)^They are used as part of screening programs together with the ultrasonographic measurement of nuchal translucency.^(11)^Differences in the levels of PAPP-A and free β-hCG are thought to possibly indicate impaired placentation,^(11)^an abnormal karyotype,^(12)^ poor obstetrics outcomes,^(4, 5,12)^and TM.^(12-15)^

As a result, threatened miscarriage,^(4,5)^subchorionic hematoma,^(9,10)^ and levels of PAPP-A and free β-hCG^(11,12)^appear to be associated with poor obstetric outcomes and suggesting placentation-related disorders.^(8)^ Although the changes in the levels of PAPP-A and free β-hCG used in first-trimester screening tests have been investigated by many researchers^(12-15)^ in cases with TM, the effect of the presence of SH in these cases has not been investigated as our knowledge. This study aimed to investigate the effect of the presence of SH on free β-hCG and PAPP-A levels measured in first-trimester maternal serum.

## Methods

**Figure 1:**
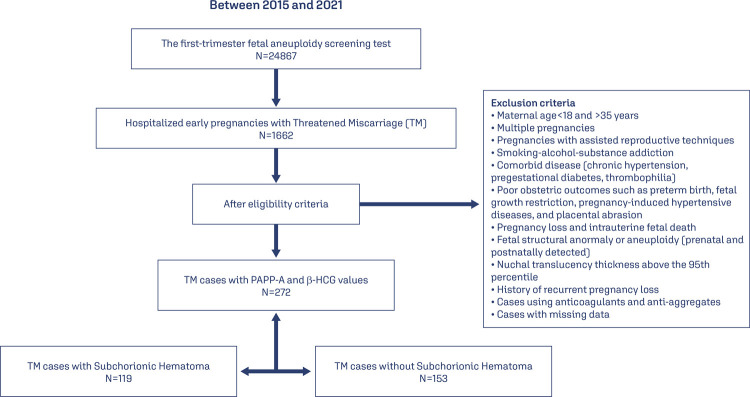
Patients selection process

In this case-control study, all cases who were hospitalized to the early pregnancy service due to TM and who had a first-trimester fetal aneuploidy screening test were scanned from the hospital registry system between 2015 and 2021 years. To evaluate the effect of the SH on the biochemical markers (PAPP-A and B-HCG) in the first-trimester fetal aneuploidy screening test, we retrospectively evaluated the data of cases diagnosed with fetal viability and SH before the first-trimester screening test and whose reached at term (> 36 weeks 6 days) in our medical center. Thus, among TM cases who were hospitalized, SHs were detected in ultrasonography reports at the time of admission, and those who obtained the results of the first-trimester fetal aneuploidy screening test were designed as the study group. To constitute the control group, data of age and gestational age-matched at the time of the first-trimester screening test of 153 patients without SH between the same time of the study period were retrospectively evaluated. The flow chart of the study is presented in figure 1.

We included the pregnancies with singleton fetuses, aged between 18 and 35 years, and those who had been not diagnosed with any poor obstetric outcomes. Multiple pregnancies, pregnancies with assisted reproductive techniques (ART), with smoking-alcohol-substance addiction, with comorbid disease (chronic hypertension, pregestational diabetes, thrombophilia), with poor obstetric outcomes as PB, PIHD, FGR, and PA, with pregnancy loss and intrauterine fetal death, with fetal structural anomaly or aneuploidy (prenatal and postnatally detected), with nuchal translucency (NT) thickness above the 95^th^ percentile, with history of recurrent pregnancy loss and cases using anticoagulants and anti-aggregates were excluded from the study.

The combination of maternal serum and ultrasound screening in the first trimester has made it possible to offer all pregnant patients a non-invasive screening test to assess the risk of a fetus with aneuploidy to determine whether invasive prenatal diagnostic testing is necessary. Ultrasonography is performed by sonographers qualified in first-trimester screening, and crown-rump length (CRL) and NT are measured in a standardized fashion (www.fetalmedicine.com). Fetal CRL is 45-85 mm in first-trimester screening, it is routinely recommended for all pregnant women in our hospital and in volunteers for testing, maternal venous blood is collected in EDTA plasma tubes at the 11th and 14th weeks of gestation. In the first-trimester fetal aneuploidy screening test, data such as NT, age, weight, ethnicity, gestational age, CRL, diabetes, and smoking status are evaluated along with serum PAPP-A and free β-hCG results in the PRISCA 5.0.2.37 (Prenatal Risk Calculation, TYPOLOG Software/GmBH, Hamburg, Germany) package program. Serum β-hCG and PAPP-A levels measured in each pregnant woman are calculated with Multiples of the Median (MoM) values using the median values determined according to the normal population in the Prisca program for the same gestational age. All laboratory methods are continuously evaluated by intra- and extra-mural quality assurance programs. As a result, we recorded the MoM values to evaluate the free β-hCG and PAPP-A levels in both groups with and without SH. All obtained data were compared in both groups. Demographic characteristics, gynecological and obstetric histories, ultrasonographic findings (gestational age, presence of SH, SH size, viability), and other detailed information of patients included in the two groups were compared.

In the power analysis conducted according to the study of De Biasio et al.,^(13)^the number of samples calculated with an effect level of 0.80, an α value of 0.05, and a power value (1-β) of 0.80 was determined to be 52. Statistical analyses were performed with the SPSS 28.0.1.0 (version 142) program at a 95% confidence level. The conformity of the variables to the normal distribution was checked with the Kolomogorov-Smirnov and Shapiro-Wilk test and no data were found to be normally distributed. All data were expressed as median (interquartile range) or number (percentage %). Categorical variables were analyzed with the chi-square test. Mann-Whitney U test was used for non-parametric numeric data. A value of 0.05 or less was accepted for the p-value, which meant a significant difference between the groups. Multivariate regression analysis was used to compare the MoM values of free β-hCG and PAPP-A with other parameters such as age, gravida, and vertical and longitudinal lengths of the hematoma.

Ethical approval was obtained from the local ethics committee for the survey with approval number 14/20 and date 17/12/2021, and the authors of the study complied with the Helsinki Declaration of Human Rights.

## Results

**Figure 2:**
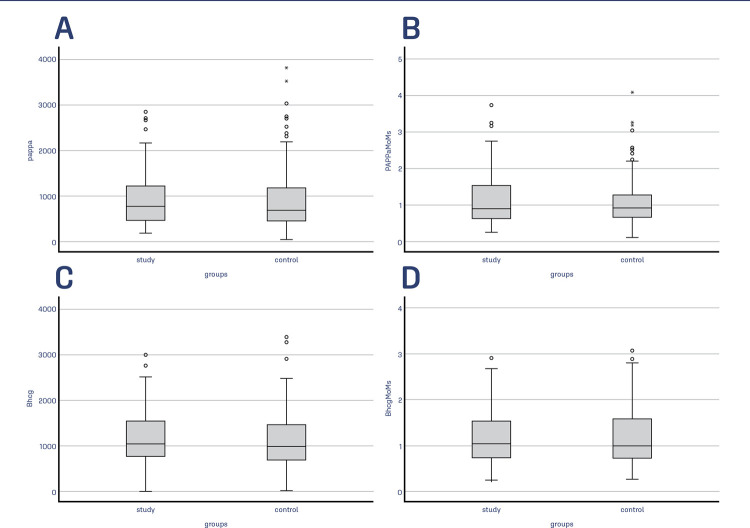
Box plot of groups according to the value of PAPP-A (Figure A), MoM of PAPP-A (Figure B), the value of Free β-hCG (Figure C), and MoM of Free β-hCG (Figure D)

There were 119 cases in the study group with SH and 153 cases in the control group without SH. In the study group, the median vertical length of the SH was 31 mm (29.25mm) and the median longitudinal length was 16 mm (17 mm) (Table 1). The median age in both groups was similar and found as 27 years (p=0.422). The numbers of gravida, parity, abortion, vaginal and cesarean delivery were similar in both groups (p>.05). In the birth type of current pregnancy, 63% of the study group delivered vaginally, and 62.2% of the control group delivered vaginally (p=.875). All pregnancies included in the study reached term, but the study group with SH delivered one week earlier than the other group (median birth week 38w vs. 39w) (p=.011). The gestational week at which TM occurred and the weeks of gestation at which the groups had the first-trimester screening test and NT thickness were also similar (p>.05). The MoM value of PAPP-A was 0.088(.93) MoM in the study group and 0.9 (.63) MoM in the control group (p=0.519). Similarly, the MoM value of Free β-hCG was 1.04 (.78) MoM in the study group and .99(.86) MoM in the control group (p=0.66) (Table 1). The comparison of the groups according to the MoMs value of PAPP-A and Free β-hCG is presented in table 2. Low (<0.5) or high (>2.5) MoM values of PAPP-A and Free β-hCG did not differ significantly between the groups (p>.05). In figure 2, box plots of the groups according to PAPP-A and Free β-hCG values and MoMs are presented.

**Table 1:** Comparison of groups according to demographic and obstetric characteristics

Variables	Groups		p-value
	Study Group subchorionic hematoma + n=119	Control Group subchorionic hematoma - n=153	
	Median (IQR)	Median (IQR)	
Age	27(8)	27(9)	0.422
Number of gravidea	2(2)	2(2)	0.186
Number of parity	1(2)	1(2)	0.081
Number of previous abortions	0(0)	0(1)	0.655
Number of previous vaginal birth	0(2)	0(1)	0.097
Number of previous cesarean birth	0(0)	0(0)	0.904
Birth week	38.0(1.25)	39.0 (1.0)	0.011*
Gestational week at TM	11.0 (3.25)	11.0 (4)	0.476
Week of gestation in screening test	12.0(1)	12.0(1)	0.330
The thickness of NT (mm)	1.20(0.40)	1.20(0.60)	0.164
PAPP-A(ng/ml)	780.0(754)	701.8(748)	0.699
PAPP-A MoM	0.88(0.93)	0.90(0.63)	0.519
Free β-hCG (mUI/ml)	104919.0(78624)	99408.0(77051)	0.350
Free β-hCG MoM	1.04(0.78)	0.99(0.86)	0.662
Vertical length of SH (mm)	31.0(29.25)	-	-
Longitudinal length of SH (mm)	16(17)	-	-

β-hCG - Free β-human chorionic gonadotropin; IQR - interquartile range; NT - nuchal translucency; SH - subchorionic hematoma; PAPP-A - Pregnancy-associated plasma protein-A; TM - threatened miscarriage Mann Whitney U Test, *p<.05

**Table 2:** Comparison of groups according to MoM value of PAPP-A and Free β-hCG

Comparison		Groups		Total	p-value
		Study Group subchorionic hematoma + n(% within groups)	Control Group subchorionic hematoma – n(% within groups)		
Free β-hCG MoM	<0.5 MoM Low	9(7.6)	9(5.9)	18(6.6)	0.369
	0.5-2.5 MoM Normal	108(90.8)	137(89.5)	245(90.1)	
	>2.5 MoM High	2(1.7)	7(4.6)	9(3.3)	
PAPP-A MoM	<0.5 MoM Low	16(13.4)	22(14.4)	38(14.0)	0.962
	0.5-2.5 MoM Normal	98(82.4)	124(81.0)	222(81.6)	
	>2.5 MoM High	5(4.2)	7(4.6)	12(4.4)	
Total		119	153	272	

β-hCG - Free β-human chorionic gonadotropin; PAPP-A - Pregnancy-associated plasma protein-A; MoM - multiple of the medians; Chi-Square test

No significant relationship was detected in the multivariate analysis between the MoM of PAPP-A and age, gravida, MoM of free β-hCG, and vertical and longitudinal lengths of the hematoma (p = 0.437, R^2^ = 0.042, aR^2^ = -0.001). Moreover, no significant relationship was detected in the multivariate analysis between MoM of free β-hCG and age, gravida, MoM of PAPP-A, and vertical and longitudinal lengths of the hematoma (p = 0.312, R^2^ = 0.051, aR ^2^= 0.009). The coefficient results of the multivariate analysis between PAPP-A MoM and free β-hCG MoM and other parameters are presented in table 3.

**Table 3:** The coefficient results of the multivariate analysis between PAPP-A MoM and free β-hCG MoM and other parameters

Model		Coefficients			t	Sig.	95.0% confidence Interval for B	
		Unstandardized coefficients		Standardized coefficients				
		B	Std. Error	Beta			Lower bound	Upper bound
PAPP-A MoM	(Constant)	1.697	0.418		4.063	<0.001	0.869	2.524
	Age	-0.014	0.014	-0.101	-1.010	0.315	-0.043	0.014
	Number of gravida	-0.015	0.051	-0.030	-0.301	0.764	-0.117	0.086
	The vertical length of subchorionic hematoma	-0.003	0.004	-0.091	-0.852	0.396	-0.011	0.004
	The longitudinal length of subchorionic hematoma	-0.004	0.005	-0.096	-0.902	0.369	-0.014	0.005
	Free β-hcg MoM	0.027	0.111	0.023	0.244	0.808	-0.192	0.246
free β-hCG MoM	(Constant)	1.600	0.351		4.560	<0.001	0.905	2.296
	Age	-0.022	0.012	-0.185	-1.870	0.064	-0.046	0.001
	Number of gravida	0.078	0.043	0.178	1.819	0.072	-0.007	0.164
	The vertical length of subchorionic hematoma	-0.002	0.003	-0.064	-0.595	0.553	-0.008	0.005
	The longitudinal length of subchorionic hematoma	0.002	0.004	0.056	0.526	0.600	-0.006	0.011
	PAPP-A MoM	0.020	0.081	0.023	0.244	0.808	-0.140	0.180

A multivariate regression analysis

## Discussion

It has been known that pregnancies with SH and with abnormal levels of B-HCG and low levels of PAPP-A are associated with a higher risk for poor obstetrics outcomes than those without it. Therefore, the effect of the presence of SH on biochemical parameters (B-HCG and PAPP-A) of the first-trimester fetal aneuploidy screening test in TM cases needs to be clarified. To our best knowledge, there has not been any study evaluating the effect of SH on the value of B-HCG and PAPP-A in the first trimester.

Some studies,^(12-16)^have found that early vaginal bleeding in the first and second trimesters of pregnancy shows an increase in maternal serum free β-hCG and explains this with disruption of the maternal-fetal interface and subsequently an increase in the rate of passage of the hormone into the maternal circulation. However, De Biasio et al.^(13)^ examined the effect of early vaginal bleeding on first-trimester markers and reported a significant 9% increase in free β-hCG, but no significant difference in PAPP-A.^(13)^Heinig et al.^(14)^ found a 10% increase in free β-hCG and a 12% increase in PAPP-A in cases with TM but did not find a significant difference in MoM values between the groups. Moreover, Spencer et al.^(15)^compared 7470 women who self-reported vaginal bleeding and 42 183 women who reported no vaginal bleeding at any stage prior to the screening test in the study, which showed that vaginal bleeding had little or no effect on first-trimester maternal serum marker levels and required no correction. As in our study, there was no significant difference between the groups by median test (p = 0.080) or by comparing log MoMs (p = 0.1305) for free beta-hCG and PAPP-A with the median test.^(15)^

Some pieces of evidence decreased maternal serum concentrations of PAPP-A and free β-hCG are associated with adverse subsequent pregnancy outcomes in pregnancies with^(17,18)^or without^(11,19,20)^a previous episode of early vaginal bleeding. Furthermore, the Royal College of Obstetricians and Gynecologists (RCOG, 2014)^(21)^ guideline states that higher ultrasound surveillance is required for growth disorders in women with serum PAPP-A <0.415 MoM (5th percentile). Low PAPP-A levels have been associated with PA, and these data have also argued that the origins of PA can be traced back to the early stages of pregnancy.^(22)^In addition, Sirikunalai et al.^(23)^ reported that lower (< 0.5 MoM) and high (> 2.0 MoM) β-hCG groups which were detected in the second trimester (n = 5470) and showed that it significantly increased poor obstetric outcomes such as spontaneous abortion, IUGR, and PB. On the other hand, it has been shown that TM^(12-15)^and SH^(9,10,24)^can affect normal placental development and are associated with adverse pregnancy outcomes. Unfortunately, the exact pathophysiology of SH is still unclear, but poor plesantation may impair angiogenesis, resulting in weak vessels that rupture easily, and low-pressure bleeding is suggested to occur in SH due to the rupture of marginal uteroplacental vessels.^(25)^

In light of this data, we investigated with great curiosity the relationship of subchorionic hematomas formed during early pregnancy with PAPP-A MoM and free β-hCG MoMs, which are among the first-trimester fetal aneuploidy screening tests. Through, abnormal processes may be associated with intrauterine abnormal placentation in early gestational weeks and these entities may affect each other. However, to our knowledge, there are not many recent studies on this subject. One of these rare studies is the study by Pedersen et al.^(26)^in 1995, according to this study, there was no difference between serum levels of human placental lactogen (hPL), PAPP-A, and endometrial secretory protein PP14 (PP14) in 29 women with and 40 women with subchorionic hemorrhage, and there was no correlation between serum levels and hematoma size. We found no significant difference in TMs with and without SH, in terms of the values of PAPP-A in ng/ml and Free β-hCG in mUI/ml, MoM values of PAPP-A and Free β-hCG, and low (<0.5) or high (>2.5) MoM values of PAPP-A and Free β-hCG. In the current study, no significant relationship was found in the multivariate analysis between free β-hCG MoM, PAPP-A MoM, age, gravida, and vertical and longitudinal lengths of the hematoma.

One of our top priorities in the design of this study, which was also the strength of the study, was the strict application of inclusion and exclusion criteria. Through, as mentioned above, many parameters affect PAPP-A MoM, and free β-hCG MoM levels in the first trimester. Not only fetal structural and chromosomal anomalies, but also pregnancies with poor obstetric outcomes were excluded, and the pure effect of the presence of subchorionic hematoma in the two groups that resulted in a normal singleton pregnancy was investigated. As a limitation, although this study is retrospective, the retrospective effect of the study and inconsistencies in the patients’ statements were excluded by including TM cases with proven diagnoses that were hospitalized in the early pregnancy service of a tertiary center with thousands of deliveries per month, and including SH cases that were definitively detected ultrasonographically in their medical records.

## Conclusion

The presence of subchorionic hematoma (SH) does not affect PAPP-A, and free β-hCG levels in the first trimester, which is one of the screening tests obtained from maternal serum in patients with threatened miscarriage (TM) in the early stages of pregnancy. Nevertheless, PAPP-A and free β-hCG can be used confidently in patients with SH as well as those without SH in the first trimester for the fetal aneuploidy screening tests. Further prospective randomized studies are still needed to determine the true role of SH in its effect on PAPP-A, and free β-hCG levels. Therefore, the presence of TM with SH should be investigated at abnormal PAPP-A MoM, and free β-hCG MoM levels, and these patients should be provided careful antenatal care to reduce the risks for adverse pregnancy outcomes until sufficient evidence is obtained.
